# Construction and implementation of a death education program for nursing interns: an action research study

**DOI:** 10.1186/s12909-025-08495-8

**Published:** 2026-01-07

**Authors:** Jinlong Liu, Xiaofan Guo, Binbin Hou, Yunjia Xu, Meirong Hong, Nuo Xu, Wei Zhang, Yun Xia, Yan Lou

**Affiliations:** 1 School of Public Health and Nursing, Hangzhou Normal University, No. 2318, Yuhangtang Rd, Yuhang District, Hangzhou, Zhejiang 311121 People’s Republic of China; 2 Nursing College, Naval Medical University, 800 Xiangyin Road, Shanghai, 200433 China; 3 Nursing Department, Sir Run Run Shaw Hospital, Zhejiang University School of Medicine, Hangzhou, China; 4 National Institute of Education, Nanyang Technological University, Singapore, Singapore; 5 Nursing Department, Hangzhou Cancer Hospital, No. 34, Yanguanxiang Rd, Shangcheng District, Hangzhou, Zhejiang 310002 People’s Republic of China

**Keywords:** Death education, Intern nursing students, Constructivist learning theory, Action research

## Abstract

**Background:**

Nursing interns frequently encounter patient death but are often inadequately prepared for end-of-life care, leading to distress and reduced efficacy. Existing death education programs rarely address their specific needs.

**Objective:**

To develop and evaluate a tailored death education program for nursing interns.

**Methods:**

The study was conducted in two phases: development and refinement of a preliminary program through literature review, interviews, Delphi method, and the first action research cycle; followed by evaluation via a second action research cycle with 48 interns (24 per cycle). Quantitative outcomes were assessed using the Death Attitude Profile-Revised (DAP-R), Frommelt Attitude Toward Care of the Dying scale (FATCOD-B), and Coping with Death Scale (CDS) scales, while qualitative data were collected through semi-structured interviews and participatory observation.

**Results:**

After the second cycle, significant improvements (*P* < 0.05) were observed across all DAP-R dimensions, FATCOD-B total score, and CDS total score. The second cycle yielded significantly greater improvements in most outcomes compared to the first (*P* < 0.05), except for Escape Acceptance (*P* = 0.073). Qualitative analysis identified three themes: reflections and recommendations on death education workshops; development of end-of-life communication skills and deepening death awareness and Internalized development of life care and empathy capacity.

**Conclusion:**

The action research-based program effectively improved nursing interns’ death coping, end-of-life care attitudes, and communication skills, supporting the integration of experiential and reflective pedagogies into palliative care education.

**Supplementary Information:**

The online version contains supplementary material available at 10.1186/s12909-025-08495-8.

## Background

Changing patterns of disease, improved healthcare access, smaller family sizes, and evolving societal attitudes have gradually shifted the primary location of death from homes to hospitals [1]. This shift increases healthcare professionals' exposure to death, making their ability to cope with death essential [2].

Nurses assume a more hands-on and enduring role in end-of-life care than physicians. Their responsibilities encompass holistic patient support—managing symptoms, aiding daily activities, and addressing psychological needs—alongside regular communication to help patients and families understand conditions, alleviate distress, and make informed choices [3]. Nurses also provide postmortem care, preserving the deceased's dignity, and deliver grief counseling to bereaved families to aid in their bereavement process [4]. This deep involvement and sustained interaction with patients and their families place nurses in complex and emotionally demanding end-of-life roles, necessitating advanced coping competencies.

Nursing interns are in a critical transitional stage between student and professional roles, during which their professional identity and clinical competencies are formed [5]. Exposure to high-mortality clinical environments—such as emergency departments, oncology units, and ICUs— positions them at the forefront of end-of-life care, where their competence in managing death directly impacts patient and family experiences [6]. With a mean age of 21, nursing interns often experience emotional distress when caring for dying patients [7]. This distress may include fear of death, anxiety during end-of-life conversations, uncertainty about how to comfort families, and concerns about making mistakes at the bedside. Qualitative studies [8, 9] further show that interns may feel helpless and panicked when caring for dying patients, and may develop sleep problems, emotional withdrawal, or even avoidance of terminally ill patients due to limited preparation before clinical practice. Such death-related emotional strain not only impedes clinical learning outcomes but also influences long-term career decisions [10], contributing to China's concerning annual nurse attrition rate of 7.7% [11].

Current evidence indicates death education effectively enhances nursing students' death coping competence and mitigates death-related distress [12]. However, significant gaps persist in death education for nursing interns. First, the specific educational needs of this group have not been fully recognized or met [13]. Second, existing research fails to adequately consider the unique developmental stage of nursing interns within their professional trajectory [14]. Third, a disconnect persists between theoretical death education and clinical practice for nursing students [15].

Although scholars have explored this field, such as Duan et al., who innovatively integrated traditional culture into death education through the Heart to Heart Tea House to improve interns' attitudes towards death [16], and Yang et al., who preliminarily developed an experiential death education program [17], these studies remain insufficient for dynamically addressing the nursing interns' needs. Consequently, there is an urgent need to develop a tailored death education system aligned with the professional development characteristics of nursing interns. This will not only help to enhance their professional competence and hospice care skills but also contribute significantly to reducing nurse turnover in high-stress medical environments.

Action research was initially applied in social psychology, aiming to integrate theory and practice into a cohesive framework [18]. Over time, its application has extended to educational contexts and sociological inquiry. By synthesizing methodologies from the natural sciences and humanities, Action research bridges a critical gap between theoretical knowledge and practical implementation [19]. Central to this approach is its iterative "Plan- Act- Observe- Reflect" cycle [20]. This process enables educational programs to dynamically adapt to the evolving needs of nursing interns, thereby enhancing pedagogical effectiveness. Moreover, the participatory characteristic of Action Research fosters collaborative engagement between learners and educators, cultivating an open learning environment conducive to critical discourse [21]. Therefore, this study employs action research as its methodological foundation. This framework provides both the scientific rationale and pragmatic methodology for developing and implementing a death education program tailored to nursing interns.

This study has two objectives: (1) to develop a death education program for nursing interns and optimize it through a first-cycle of action research; and (2) to evaluate the program's effectiveness via a second-cycle action research.

## Methods

### Research design

We developed a death education program for nursing interns grounded in constructivist learning theory and refined it through two iterative cycles of action research. The choice of action research was strategic, as it allows for the real-time, classroom-based refinement of pedagogical strategies through direct observation and evidence-driven adjustments. The entire process, governed by the "Plan-Act-Observe-Reflect" spiral, was executed by a core team with distinct responsibilities:

Plan (Program Development & Refinement): The preliminary program was developed by the principal researcher (LJL) and the educational designer (LY) through literature review, expert consultation (Delphi method), and initial needs assessment. The refinement of the program for the second cycle was a collaborative effort led by LJL, LY, and the co-researcher (GXF), based on quantitative and qualitative findings from the first cycle.

Act (Program Implementation): The death education workshops were facilitated by the principal researcher (LJL) and the co-researcher (GXF).

Observe (Data Collection): Data collection during the interventions was managed by LJL and GXF, who administered the quantitative scales and conducted the semi-structured interviews and participatory observations.

Reflect (Data Analysis & Interpretation): The research team engaged in collective sense-making. LJL and GXF performed initial quantitative and qualitative data analysis. These findings were then discussed in reflective meetings involving the broader team (including LY and other co-authors) to derive insights and plan for the next cycle. This clear division of labor ensured the integrity and coherence of the action research process.

### Participants

Nursing interns were recruited from five tertiary hospitals in Hangzhou using a convenience sampling method. The recruitment process was initiated by the hospital internship coordinators (XY), who distributed the study information and invitation through internal communication channels, such as internship WeChat groups or hospital bulletins. Interested interns were then screened for eligibility based on the predefined criteria.

The sample size was determined a priori using G*Power 3.1 software based on a paired t-test parameters (effect size = 0.651, two-tailed α = 0.05, power = 0.80), yielding a minimum requirement of 21 participants. Accounting for a potential 10% attrition rate, a total of 24 interns per cycle were recruited for the death education interventions across the two action research phases. Inclusion criteria were: (1) active clinical internship status, (2) voluntary participation, (3) in good health status, (4) no prior death education training within the preceding two months, and (5) educational level: associate degree or higher. Exclusion criteria were: (1) absence during the study period (e.g., sick leave) and (2) withdrawal due to physical or psychological reasons. To mitigate potential coercion arising from the teacher-student relationship between the research team and the interns, we explicitly assured all participants that their decision to participate or withdraw would not affect their academic grades, internship assessments, clinical assignments, or their relationship with the faculty.

### Procedures

#### Design the death education program

This death education program was constructed based on constructivist learning theory. By incorporating key learning elements such as situation creation, collaboration, dialogue, and meaning construction, the program provided a safe and supportive environment. This environment enabled nursing interns to deeply understand the essence of death, accept the inevitability of life's end, and attain psychological peace and satisfaction. It also facilitated the construction of a deep understanding of death education concepts. The initial development of the program involved three steps: a literature review to clarify teaching methods, semi-structured interviews to identify the elements of end-of-life elements, and the Delphi method to refine the program's scientific basis and feasibility.

The Delphi method was employed with a panel of 13 experts, including 3 palliative care specialists, 2 oncology nursing specialists, 1 psychologist, 4 nursing educators, and 3 senior clinical nursing supervisors. The two-round Delphi process achieved a high expert consensus. In the first round, all 13 experts (100% response rate) provided feedback, resulting in a high authority coefficient (Cr = 0.91). The Kendall's W values for the importance and feasibility of the items were 0.336 and 0.337, respectively (*P* < 0.01), with coefficients of variation (CV) ranging from 0 to 0.12, indicating acceptable coordination of opinions. Based on these quantitative results and the qualitative expert feedback, specific revisions were implemented. These included refining participant exclusion criteria, re-sequencing modules to begin with film-viewing before mindfulness exercises, introducing new discussion topics on life priorities, and redesigning case scenarios to enhance role immersion and better align with the principle of patient autonomy in end-of-life decision-making. In the second round, these revisions were presented to the expert panel, which led to a final consensus rate of 85% on all components, finalizing the intervention program.

Subsequently, a two-cycle action research study was conducted among nursing interns following the "Plan-Act-Observe-Reflect" framework, with 24 students participating in each cycle. The first cycle focused on program refinement, while the second assessed its effectiveness. The refinement process was directly informed by the first-cycle findings. For instance, quantitative results from Cycle 1, while showing improvement, indicated less-than-optimal gains in "Approach Acceptance" (DAP-R) and specific FATCOD-B dimensions. This, coupled with qualitative feedback highlighting a need for more immersive role-plays and structured communication aids, led to key revisions: (1) increasing role-play preparation time, (2) broadening the use of Heart to Heart Cards across all case simulations, and (3) enhancing facilitator guidance for the Gibbs' reflective cycle. The specific time allocation for each workshop component, as presented in the final program (Table 1), was initially determined through expert consensus in the Delphi process and was subsequently validated for its practical feasibility during the implementation of Cycle 1. We conducted the first cycle of action research to optimized the death education program; the improved version is presented in Table 1. Results of the Cycle 1 action research are presented in Appendix A.

**Table 1:** The optimized death education program

Theme/Timing	Method	Specific Intervention Steps	Time Allocation	Duration	Format
Scenario Introduction (Workshop 1)	①Mindfulness Training	①Guide mindful breathing via audio; verbally prompt imagination of end-of-life scenarios	10 min	45 min	In-person (2-person groups)
	②Death Education Film	② Pose guiding questions (e.g., "When did you first encounter death?"); play "Learning to Accept Death" video; facilitate film discussion and reflection	20 min		
	③Group Discussion	③ Facilitate small group discussions on: (a) Personal experiences with death ("My Story with Death"); (b) Strategies for honest patient communication (avoiding deception); (c) Sharing personal or clinical experiences of loss	15 min		
Scenario Simulation (Workshop 1)	①Case Introduction	① Distribute case materials/scripts 1 day prior; allow 10 min for preparation immediately before role-play	10 min	60 min	In-person (2-person groups)
	②Role-Play	② Conduct sequential role-plays of 3 cases: (a) Grandma Li's Cancer Journey; (b) Rekindling Hope: Aunt Zhang's Hospital Experience; (c) Xiao Zhu's Growth: Protecting Mr. Li's Dignity & Warmth	50 min		
Scenario Reflection (Workshop 1)	Gibbs Cycle Group Reflection	Facilitate structured reflection using the Gibbs Cycle framework (Description, Feelings, Evaluation, Analysis, Conclusion, Action Plan) on simulation performance	40 min	40 min	In-person (2-person groups)
Meaning Exploration (Workshop 2)	①Mindfulness Training	① Lead mindful breathing audio for emotional regulation/focus; provide a brief recap of Workshop 1	10 min	50 min	In-person (2-person groups)
	②Heart to Heart Tea House	② Introduce the Heart to Heart cards activity; guide card selection; facilitate discussion on choices using the cards to prompt deep dialogue exploring life's meaning/value and attitudes toward death	40 min		

#### Implementing the death education program through second-cycle action research

Given the relatively short intervention period, constrained by the demanding schedules of clinical internships, the program was designed as an intensive, time-limited workshop. The death education program was implemented through small-group workshops, with each session conducted for one pair of interns (2 interns per group) at a time. These sessions were scheduled during the interns' clinical shifts in coordination with their respective hospital departments, taking place during pre-arranged, quieter periods to avoid disrupting normal ward workflow. The complete program consisted of two separate workshops (Workshop 1 and Workshop 2) with a total duration of 195 min. (1) Scenario introduction: Mindfulness breathing exercises and video-stimulated discussions on death acceptance and clinical experiences; (2) Scenario simulation: Role-playing clinical cases using Heart-to-Heart Cards to explore "good death" concepts and patient life reviews; Within each pair, interns alternated played the roles of nurse and patient/family member for different cases, allowing each participant to experience multiple perspectives. The pairs then engaged in guided discussions about their interaction. (3) Scenario Reflection: Facilitated Gibbs' reflective cycle discussions analyzing emotional responses and coping strategies; (4) Meaning Sublimation: Heart to Heart-Tea-House Tea House activities and guided dialogues on life values and mortality perspectives, concluding with program evaluation.

### Instruments

#### Demographic questionnaire

The demographic questionnaire collected information on gender, education level, ethnicity, religious affiliation, end-of-life care experience, and clinical rotation experience (e.g., oncology, ICU, geriatrics, or emergency departments). These departments were selected because they frequently involve encounters with critically ill or dying patients, making them the primary clinical settings where nursing interns are most likely to face end-of-life communication and care. The inclusion was determined through a preliminary needs assessment involving curriculum coordinators and clinical instructors during the program design phase.

#### Death Attitude Profile-Revised (DAP-R)

The DAP-R was developed by Wong et al. (2015) to assess individuals' attitudes towards death[22]. The Chinese version has demonstrated acceptable content and construct validity in previous validation studies, with a factor structure consistent with the original scale [23]. The DAP-R is a 32-item Likert-type scale comprising five dimensions of death attitudes: fear of death, death avoidance, neutral acceptance, approach acceptance and escape acceptance. A higher score indicates a stronger endorsement of the corresponding attitude towards death. In this study, the Cronbach's α coefficient was 0.88.

#### Frommelt Attitude Toward Care of the Dying scale (FATCOD-B)

The FATCOD-B, developed by Frommelt [24] in 1989, measures healthcare professionals' attitudes toward providing hospice care, including nurses and medical students. The Chinese version comprises 29 items across 6 dimensions, using a 5-point Likert scale ranging from "strongly disagree" (1) to "strongly agree" (5). Higher scores indicate more positive attitude toward hospice care. In this study, the scale demonstrated a Cronbach's α coefficient of 0.796, with a test–retest reliability of 0.959 and a content validity index of 0.92.

#### Coping with Death Scale (CDS)

The CDS was used to measure coping with death [25]. This 30-item scale employs a 7-point Likert scale ranging from "do not agree at all" (1) to "agree completely" (7). The total score is the sum of all item scores. Scores below 105 indicate inadequate coping ability, scores between 105 and 157 indicate moderate coping ability, and scores above 157 indicate optimal coping ability [26]. The Cronbach's α for the Chinese version in a previous study was 0.88 [27]. In this study, the Cronbach's α was 0.76, demonstrating adequate internal consistency reliability. The Chinese CDS has also shown satisfactory validity in earlier research, supporting its factor structure and suitability for Chinese nursing groups [27]. We used the validated version without conducting a new factor analysis.

#### Semi-structured interview and observation

We conducted one-on-one semi-structured interviews to explore participants' learning experiences, perceived benefits, and suggestions for improvement. The interview guide included the following questions: (1) What specific developments did you gain from this workshop, and what was your most significant learning outcome? (2) What are your overall impressions? (3) What are your perceptions of the teaching content and methods, and which components require further improvement? Additionally, participant observation was employed to assess nursing interns' engagement throughout the death education program. Researchers documented the implementation process, focusing specifically on: (a) program feasibility (e.g., timing, venue, and scenario arrangements) and (b) participant behaviors (including immersion in mindfulness practice, participation in film-based discussions and role-plays, and quality of reflective discussions).

### Data collection

Questionnaire data were collected electronically using Questionnaire Star (a web-based survey platform) [28]. A QR code linking to the questionnaires was provided to participants for scanning and completion. Data collection occurred at two time points: pre-intervention (before the course) and post-intervention (after the course). All data were stored securely within the platform's backend. Access to the platform account was restricted to LJL and GXF, who were solely responsible for account credentials.

Semi-structured interviews and participatory observations were conducted specifically during and after the second cycle of action research to evaluate the optimized program's effectiveness and participant experiences. Semi-structured interviews were conducted individually by two researchers (LJL and GXF), both postgraduate nursing students trained in qualitative research methodology with no prior relationship to participants. Before each interview, researchers reiterated the study procedures and participants' rights, followed by questions based strictly on the interview guide. At the conclusion, researchers confirmed whether participants had additional comments and obtained permission for potential future contact. All interviews, lasting 40–60 min, were audio-recorded. Verbatim transcription was performed by the first author within 24 h of each interview session.

Additionally, participant observation sessions were video-recorded to document participants' conversations, behaviors, and interaction patterns during each thematic workshop activity.

### Data analysis

Quantitative data were analyzed using SPSS 19.0 software [29]. Normally distributed continuous variables are presented as mean ± standard deviation (SD). Paired t-tests were used for pre-post comparisons. Categorical data are presented as frequencies and percentages (%). Statistical significance was set at *P* < 0.05.

For qualitative data, thematic content analysis was employed [30], a method particularly suited for validating, refining, or extending theoretical frameworks in new contexts. Data collection and analysis occurred concurrently. First, two researchers (LJL and GXF) repeatedly reviewed the transcripts to achieve immersion and develop a general understanding of interns' perspectives on 'good death'. Subsequently, the first author (LJL) performed initial open coding. These codes were independently reviewed by a second researcher (GXF); disagreements were resolved through joint re-examination of the original interview text. Next, the first author (LJL) organized categories and main themes, supported by illustrative participant quotes. Throughout this process, researchers critically examined participants' core messages to deeply understand interns' learning experiences, perceived gains, and program recommendations. Finally, all authors engaged in iterative discussions of the coded data until consensus was reached. To enhance rigor, the coding and theme development process followed Braun and Clarke's six-phase framework for reflexive thematic analysis [31], incorporating analyst triangulation and consensus meetings. The entire analytical process was conducted in Chinese, with the final results translated into English for reporting.

### Ethical consideration

This study adhered to the ethical principles outlined in the Declaration of Helsinki for medical research involving human subjects. Ethical approval (Approval No.: 2024082) was obtained from the Ethics Committee of Hangzhou Normal University School of Public Health and Nursing prior to study commencement following formal review. Participants received comprehensive written information regarding study aims, procedures, and anonymization measures before providing written informed consent. All data were securely stored, with confidentiality strictly maintained, and used exclusively for research purposes. To prevent any risk of workplace retaliation, participation or withdrawal had no impact on academic evaluations, internship assessments, clinical assignments, or relationships with teaching faculty. Recruitment and data collection were conducted independently of clinical supervisors, and the research team had no administrative authority over participants.

## Results

### Demographic characteristics

All 24 interns recruited for the second action research cycle completed both the intervention and the post-test assessments, resulting in no attrition and a final analysis sample of 24. The participants had a mean age of 21.5 years (range: 21–22 years). The cohort was predominantly female (21 out of 24, 87.5%), reflecting the typical gender distribution among nursing interns in China. A comparison of baseline demographic characteristics between the participants in the first (*n* = 24) and second (*n* = 24) action research cycles revealed no statistically significant differences (all *P* > 0.05), confirming the comparability of the two groups at baseline. Their complete demographic characteristics are presented in Table 2.

**Table 2:** Demographic characteristics

Item	Options	First Action Research Cycle (*n* = 24)		Second Action Research Cycle (*n* = 24)		*χ*^*2*^	*P*
		Frequency	Percentage (%)	Frequency	Percentage (%)		
Gender	Male	2	8.33	3	12.5	0.223	0.637
	Female	22	91.67	21	87.5		
Age	21	9	37.5	11	45.83	0.343	0.558
	22	15	62.5	13	54.17		
Academic qualifications	Undergraduate students	22	91.67	20	83.33	0.762	0.383
	Associate degree students	2	8.33	4	16.67		
Ethnicity	Han	23	95.83	24	100	1.021	0.312
	Manchu	1	4.17	0	0		
Experience of family member's passing away	Yes	12	50	8	33.33	1.371	0.242
	No	12	50	16	66.67		
Frequency of contact with dying patients	Never	7	29.17	2	8.33	5.068	0.167
	Rare	14	58.33	17	70.83		
	Sometimes	3	12.5	3	12.5		
	Often	0	0	2	8.33		
Experience in oncology, ICU, geriatrics, emergency department	Yes	10	41.67	11	45.83	1.333	0.248
	No	14	58.33	13	54.17		
Formal death education	Yes	7	29.17	11	45.83	1.422	0.233
	No	17	70.83	13	54.17		

### Quantitative research results

#### Attitudes toward death

Post-intervention, nursing interns showed statistically significant improvements (*P* < 0.05) in all five death attitude dimensions—Fear of Death, Death Avoidance, Neutral Acceptance, Approach Acceptance, and Escape Acceptance—compared to baseline (Table 3). The magnitude of improvement was significantly greater (*P* < 0.05) in Cycle 2 versus Cycle 1 for all dimensions except Escape Acceptance (*P* = 0.073) (Table 4).

**Table 3:** DAP-R scores before and after the second cycle of action research (*n* = 24)

	Pre-teaching	Post-teaching	*t*	*P*
Fear of death	22.00 ± 2.43	13.38 ± 2.39	11.826	< 0.001***
Death avoidance	15.50 ± 3.20	8.63 ± 1.38	9.967	0.001**
Neutral acceptance	16.54 ± 1.74	21.79 ± 1.35	−15.359	< 0.001***
Approach acceptance	26.92 ± 3.59	34.13 ± 3.85	−5.377	0.001**
Escape acceptance	12.67 ± 2.85	16.92 ± 3.80	−3.927	0.001**

Footnote: *DAP-R* Death Attitude Profile–Revised

^*^P < 0.05

^**^P < 0.01

^***^P < 0.001

**Table 4:** Comparison of the difference in DAP-R between the two groups

	Groups		*t*	*P*
	First cycle of action research (*n* = 24)	Second cycle of action research (*n* = 24)		
Fear of death	−3.50 ± 6.03	−8.63 ± 3.57	3.583	0.001**
Death avoidance	−3.38 ± 4.75	−6.88 ± 3.38	2.94	0.005**
Neutral acceptance	2.63 ± 2.10	5.25 ± 1.67	−4.785	< 0.001***
Approach acceptance	3.04 ± 6.47	7.21 ± 6.57	−2.214	0.032*
Escape acceptance	1.17 ± 6.32	4.25 ± 5.30	−1.832	0.073

Footnote: *DAP-R* Death Attitude Profile–Revised

^*^P < 0.05

^**^P < 0.01

^***^P < 0.001

#### Attitudes toward end-of-life care

In Cycle 2, significant improvements (*P* < 0.05) occurred in hospice care attitude total scores pre- versus post-intervention (Table 5). These improvements were significantly greater (*P* < 0.05) than those achieved in Cycle 1 (Table 6).

**Table 5:** Comparison of FATCOD and CDS scores before and after the second cycle of action research (*n* = 24)

Scale	Pre-teaching (Mean ± SD)	Post-teaching (Mean ± SD)	*t*	*P*
FATCOD	91.96 ± 6.72	117.04 ± 8.43	− 12.979	0.001**
CDS	124.33 ± 15.52	172.50 ± 10.29	− 12.659	0.001**

Footnote: *FATCOD* Frommelt Attitude Toward Care of the Dying Scale, *CDS* Coping with Death Scale

^*^P < 0.05

^**^P < 0.01

^***^P < 0.001

**Table 6:** Comparison of pre-post change scores in FATCOD and CDS between the two action research cycles (*n* = 24)

Scale	Groups		*t*	*P*
	first cycle of action research	second cycle of action research		
FATCOD	9.88 ± 12.93	24.21 ± 8.24	−4.581	< 0.001***
CDS	23.58 ± 22.04	48.17 ± 18.64	−4.172	< 0.001***

Footnote: *FATCOD* Frommelt Attitude Toward Care of the Dying Scale, *CDS* Coping with Death Scale

^*^P < 0.05

^**^P < 0.01

^***^P < 0.001

#### Death coping competence

Significant increases (*P* < 0.05) in death coping competence scores were observed post-intervention in Cycle 2 (Table 5). This improvement was significantly greater (*P* < 0.05) than in Cycle 1 (Table 6).

### Qualitative research results

At the end of the second cycle of action research, the researcher collected qualitative feedback from the research participants based on the interview outline. The interview groups in the second cycle of action research were numbered N1-N8.

#### Theme 1: Reflections and recommendations on death education workshops

##### Subtheme 1.1: Providing a relaxed atmosphere for comfort and engagement with sensitive topics

The workshop employed diverse interactive methodologies to facilitate engagement with the sensitive topic of death within a supportive environment. While initial discomfort was reported due to topic sensitivity, the open and relaxed atmosphere that was gradually established effectively mitigated psychological barriers, enabling active participation.

This workshop was completely different from any traditional course I've taken! Instead of dull lectures, we explored death through engaging activities like role-playing, group discussions, and video sharing. Initially uncomfortable with the heavy topic, I gradually felt at ease in the welcoming atmosphere and became fully engaged in the discussions. (N2).

##### Subtheme 1.2: Recommendations for workshop improvement

Participants prioritized improvements to the "Scenario Simulation" and "Meaning Sublimation" modules, specifically recommending: (1) broader implementation of Heart to Heart Cards across all case studies; (2) incorporation of authentic hospice care documentaries immediately preceding simulations to foster quicker engagement; and (3) strategic involvement of death education specialists, clinical instructors, or patients in Heart to Heart Tea House sessions.

Incorporating the Heart to Heart Cards in more cases would strengthen interactivity and immersion. (N8)

Showing real documentaries before simulations would help us better understand actual clinical contexts. This preparation would make role-playing feel more natural and improve our responses. (N5)

Having death education specialists, instructors, or actual patients join our Heart to Heart Tea House discussions would be incredibly beneficial! Their diverse perspectives and experiences would make sessions more meaningful and educational. (N1).

#### Theme 2: Development of end-of-life communication skills and deepening death awareness

##### Subtheme 2.1 Enhancing interns' communication competence in end-of-life care through practical and compassionate interaction strategies

Three distinct end-of-life case studies provided practical guidance for diverse clinical communication needs, enhancing interns' understanding of patient-family dynamics. The workshops concurrently improved nonverbal communication proficiency, with participants demonstrating developed skills in compassionate body language and eye contact. Observations revealed that introverted participants employed alternative methods for expressing empathy and providing support: one used drawings to explain a patient's condition (Auntie Zhang case), while another incorporated subtle acts of care—such as adjusting bedding and offering tissues (Nurse Zhu case). Additionally, interns developed competency in using Heart to Heart Cards to sensitively elicit families' sharing of patients' unfulfilled wishes while maintaining emotional regulation during emotionally charged interactions.

These case dialogues were extremely practical—like giving us communication "templates". Each case presented different scenarios, like dealing with calm patients or emotionally distraught family members. They help us anticipate how to respond. Now when I encounter similar situations in clinical practice, I'll know how to initiate conversations without panicking. (N3).

During role-playing, I learned to express empathy through gestures and eye contact. For instance, when simulated patients expressed death anxiety, I would gently hold their hands to show understanding and attentiveness. (N1).

In the simulation, I used Heart to Heart Cards to guide my peer (playing a family member) to share the patient's unfinished wishes. This experience taught me how to remain calm and communicate effectively in highly sensitive situations. The key lesson was that genuine listening is essential before any meaningful response or support. (N7).

##### Subtheme 2.2: Deepening comprehensive understanding of death through role-playing and group discussion

Dual-role simulations (patient/nurse perspectives) and exposure to personal narratives enabled participants to empathize with patients' mortality experiences while acknowledging death as an inherent to human existence. Concurrently, structured group discussions expanded their understanding of spiritual needs beyond religious frameworks to encompass existential meaning-making processes.

The simulation where I played both patient and nurse roles gave me profound insight into patients' emotional struggles when facing mortality. (N1)

During discussions, a peer's insight that spiritual needs include existential meaning-seeking, not just religious aspects, fundamentally deepened my understanding(N6).

#### Theme 3: Internalized development of life care and empathy capacity

##### Subtheme 3.1: Immersion and perspective-taking enhance empathy

Through immersive role-playing activities that required adopting the perspectives of both patients and family members, participants reported cultivating a deeper understanding of patients' emotional experiences and needs.

Initially, I felt quite nervous since we were simulating real-life scenarios. But once I fully immersed myself in the role, I began consciously considering the patient's and family's viewpoints. When playing a terminal patient, I asked myself: What would I truly feel if this were me? Would I be afraid? Worried about my family? What unfinished business would trouble me? This mental exercise helped me better comprehend patients' emotions and identify their needs more accurately. (N5).

##### Subtheme 3.2: Facilitating reflection on life's meaning and enhancing altruism through the heart to heart tea house

Guided narrative exchanges during the Heart to Heart Tea House fostered a shared awareness of life's transience and value, motivating participants to critically evaluate their own existential purpose. This experience concurrently cultivated altruistic commitment among interns, as they recognized their capacity as healthcare providers to meaningfully impact others' end-of-life journeys. Additionally, observations indicated that card-based interpretations elicited enthusiastic perspective-sharing among participants.

Honestly, I'd never seriously considered life's meaning before this workshop. But sharing stories and reflections during the tea session suddenly made me aware of how fleeting life is. Now I keep asking myself: What kind of life do I really want? Am I making each day count? (N6).

I originally chose nursing mainly for job security. But this workshop showed me how profoundly we can help people through this profession—caring for those who are suffering has given my life real purpose. There's incredible value in making a difference. (N8).

## Discussion

### Action research can scientifically construct and improve death education program

This study adopted an iterative action research framework based on the "Plan – Act – Observe – Reflect" spiral model, which was carried out over two cycles to systematically develop and optimize a death education program. In the first cycle, the program was initially developed and preliminarily evaluated for effectiveness. The second cycle then focused on refining the program by addressing issues identified previously. Through this dynamic, iterative process of "practice – reflection – improvement" [32], we were able to pinpoint key challenges in implementation and formulate targeted strategies for improvement, leading to positive outcomes. Quantitative findings indicated enhancements in participants' death coping competence, attitudes toward hospice care, and perceptions of death following the second cycle, underscoring the benefits of the refinements. Qualitative feedback further revealed that students experienced deeper emotional processing, increased confidence in communication, and clearer role cognition after the program was revised. In comparison with earlier action research studies in nursing [32, 33], this study strengthened the evaluation framework by integrating participatory observation with both quantitative and qualitative methods. This mixed-methods design placed central emphasis on the subjective experiences and practical needs of nursing interns, thereby enhancing the program's real-world applicability and impact. The results, particularly the more pronounced pre-post improvements seen in the second cycle across most quantitative indicators, attest to both the scientific soundness and the practical value of adopting action research in death education program development. This approach offers a replicable model for driving curricular innovation in nursing education.

### Improvement in death coping competence, communication skills, and attitudes toward death among intern nursing students

This study demonstrated that the two-cycle action research approach effectively enhanced nursing interns' death coping competence, end-of-life communication skills, and attitudes toward death. The significant reduction in scores on the “Fear of Death” and “Death Avoidance” dimensions (DAP-R) is particularly noteworthy. This quantitative shift suggests that the program helped to lower the primary psychological barriers that often prevent nursing interns from initiating end-of-life conversations. In clinical practice, this could translate into a greater willingness to approach dying patients and their families, a crucial first step in providing compassionate hospice care. This change is likely attributable to the program's immersive role-playing components, which allowed interns to safely confront and process these fears in a simulated environment [17, 34]. The significant improvement in the total FATCOD-B score underscores a more positive attitude toward hospice care overall. This quantitative finding is vividly illustrated by the qualitative theme “Development of end-of-life communication skills”. For instance, one intern's remark, “*Now when I encounter similar situations in clinical practice, I'll know how to initiate conversations without panicking (N3)*,” provides a narrative explanation for the improved FATCOD-B scores. It demonstrates how the acquired practical skills directly contributed to building a more confident and proactive care attitude.

In the thematic components "Scenario Introduction" and "Scenario Reflection," open-ended questions and real clinical dilemmas were used to stimulate critical thinking and guided discussion [35]. This method not only promoted a deeper understanding of death-related issues but also strengthened students' abilities in emotional expression and articulating their viewpoints. The "Scenario Simulation" component innovatively combined case analysis with role-playing using Heart to Heart cards. These cards visually represent patient needs across physiological, psychological, social, and spiritual domains, offering a structured communication framework [36]. By serving as a conversational anchor or "buffer zone," the cards helped students focus on the patient narratives depicted, thereby mitigating initial communication barriers, such as anxiety about what to say, and supporting more natural end-of-life dialogues. Moreover, students reported gaining experience in managing common death-related clinical situations from multiple role perspectives, aligning with findings by Yoong et al. [37].

The "Meaning Sublimation" component proved especially impactful. Mindfulness training helped establish a safe and open environment, effectively lowering students' psychological resistance to death-related topics [38]. Concurrently, the Heart to Heart Tea House format provided an interactive platform for in-depth exploration of life-and-death issues [16]. Unlike the well-known Death Café model [39], the Heart to Heart Tea House used card selection as its core guiding mechanism, enabling more structured and focused discussions on death. Within this setting, students freely exchanged perspectives and feelings about death in a relaxed and psychologically supportive atmosphere. This process was associated with a self-reported reduction in death avoidance and more open discussions about death, as evidenced by qualitative responses and improved scores on the DAP-R.

### Enhancement of nursing students' hospice care attitudes and humanistic competencies

This study found that the death education program significantly enhanced nursing interns' attitudes toward hospice care and strengthened their humanistic care competencies. These improvements can be attributed to three main factors: First, by sharing both clinical experiences in caring for terminally ill patients and personal encounters with death, the interns developed a deeper understanding of hospice care, which contributed to positive shifts in their attitudes—a finding consistent with the work of Jiang et al. (2024) [6]. Second, the use of authentic end-of-life scenarios, designed in line with the situational learning principles of constructivist theory [40], provided an immersive learning environment. Qualitative feedback indicated that this contextualized approach effectively enhanced students' empathy and perspective-taking abilities. Qualitative feedback indicated that the immersive scenarios and emotional engagement in role-playing were perceived by participants as key factors in enhancing their attitudes toward hospice care. Third, during the Heart to Heart Tea House sessions, interns engaged in guided narrative exchanges that prompted them to reflect deeply on the meaning of life and their professional identity. Through sharing personal stories and listening to peers, many recognized the profound impact they could make as healthcare providers in supporting patients at the end of life, which strengthened their sense of purpose and altruistic commitment. These exchanges encouraged students to reflect deeply on hospice care and humanistic values. Many participants expressed a heightened awareness of the importance of empathy and communication in end-of-life care, suggesting a potential shift in their professional identity [41].

### Limitations of this study

Several limitations in this study should be considered when interpreting the results. First, the relatively small sample size, recruited exclusively from Hangzhou, may limit the generalizability of the findings to other regions, cultures, or educational contexts. Second, the short duration of the intervention, dictated by clinical rotation schedules, did not allow for longitudinal assessment of attitudinal changes, which typically develop over a longer period. Third, although multiple self-reported scales were used to ensure comprehensive evaluation, they may have placed a considerable time burden on participants, potentially affecting response quality. Fourth, the lack of a control group was due to practical and ethical considerations, including the commitment to provide standard education to all interns from the same cohort and logistical difficulties in randomizing students within shared clinical environments. This limitation restricts the strength of causal inferences regarding the program’s efficacy. Fifth, it is important to acknowledge the potential for researcher subjectivity inherent in the action research approach, as the core team was directly involved in both facilitating the intervention and collecting/evaluating data. Although strategies like data triangulation and team debriefings were employed to mitigate this, the researchers' dual roles may have influenced the interpretation of qualitative findings. Furthermore, the resource-intensive nature of the program, featuring small-group workshops and specialized materials such as Heart to Heart Cards, may challenge its feasibility and scalability in resource-constrained educational settings. Future research should incorporate multicenter randomized controlled trials with extended follow-ups, examine the program's adaptability across diverse contexts, involve researchers with varied backgrounds to further reduce potential bias, and develop strategies to reduce participant assessment burden.

## Conclusion

This study systematically developed and refined a death education program for nursing interns to address critical gaps in death coping competence and prevalent negative emotions surrounding end-of-life care. Through two iterative cycles of action research, the program demonstrated significant efficacy in enhancing participants' death-related coping skills, deepening their understanding of life's meaning and death's essence, and improving humanistic care competencies alongside clinical communication skills. These findings provide valuable empirical support for optimizing death education and hospice care training curricula.

## Supplementary Information

Supplementary Material 1.

## Data Availability

The datasets generated during and/or analysed during the current study are available from the corresponding author on reasonable request.

## References

[1] Jing J, Yuan ZY. Dying in hospital versus dying at home: a sociological analysis on the location of death among Chinese citizens. Thought. 2016;42(2):14–8.

[2] Lin X, Li X, Bai Y, Liu Q, Xiang W. Death-coping self-efficacy and its influencing factors among Chinese nurses: a cross-sectional study. PLoS One. 2022;17(9):e274540. 10.1371/journal.pone.0274540 PMC9467326 36094947

[3] Kryworuchko J, Strachan PH, Nouvet E, Downar J, You JJ. Factors influencing communication and decision-making about life-sustaining technology during serious illness: a qualitative study. BMJ Open. 2016;6(5):e10451. 10.1136/bmjopen-2015-010451 PMC4885276 27217281

[4] McMillen RE. End of life decisions: nurses perceptions, feelings and experiences. Intensive Crit Care Nurs. 2008;24(4):251–9. 18162401 10.1016/j.iccn.2007.11.002

[5] Wang AH, Lee CT, Espin S. Undergraduate nursing students’ experiences of anxiety-producing situations in clinical practicums: a descriptive survey study. Nurse Educ Today. 2019;76:103–8. 30776531 10.1016/j.nedt.2019.01.016

[6] Jiang J, Zhou J, Chen X, Zhu X, Zhang H, Zhang Q, et al. The impact of clinical internship experience on nursing students’ attitudes towards death and choices of end-of-life care: a self-control study. Nurs Open. 2024;11(7):e2214. 38943259 10.1002/nop2.2214 PMC11213817

[7] Moore KA, O Brien BC, Thomas LR. I wish they had asked”: a qualitative study of emotional distress and peer support during internship. J Gen Intern Med. 2020;35(12):3443–8. 32232665 10.1007/s11606-020-05803-4 PMC7728891

[8] Wang Y, Huang Y, Zheng R, Yue X, Dong F. Intern nursing students’ perceived barriers to providing end-of-life care for dying cancer patients in a death taboo cultural context: a qualitative study. Asia Pac J Oncol Nurs. 2023;10(4):100210. 37159608 10.1016/j.apjon.2023.100210 PMC10162947

[9] Okcin FA. Examination of life and death perceptions of internship nursing students with experience of caring for unconscious patients: a qualitative study. Int J Caring Sci. 2021;14(1):745–52.

[10] Henoch I, Melin-Johansson C, Bergh I, Strang S, Ek K, Hammarlund K, et al. Undergraduate nursing students’ attitudes and preparedness toward caring for dying persons–a longitudinal study. Nurse Educ Pract. 2017;26:12–20. 28648955 10.1016/j.nepr.2017.06.007

[11] Lai P. Causes and countermeasures for the loss of nursing staff in primary hospitals. China Health Ind. 2015;12(28):17–8.

[12] Miller-Lewis L, Tieman J, Rawlings D, Sanderson C, Parker D. Correlates of perceived death competence: what role does meaning-in-life and quality-of-life play? Palliat Support Care. 2019;17(5):550–60. 30665475 10.1017/S1478951518000937

[13] Wang Y, Tang S, Hu X, Qin C, Khoshnood K, Sun M. Gender differences in attitudes toward death among Chinese college students and the implications for death education courses. Omega (Westport). 2022;85(1):59–74. 32580650 10.1177/0030222820934944

[14] Cadavero AA Jr, Sharts-Hopko NC, Granger BB. Nurse graduates’ perceived educational needs after the death of a patient: a descriptive qualitative research study. J Contin Educ Nurs. 2020;51(6):267–73. 32463900 10.3928/00220124-20200514-06

[15] Willemsen AM, Mason S, Zhang S, Elsner F. Status of palliative care education in Mainland China: a systematic review. Palliat Support Care. 2021;19(2):235–45. 32838818 10.1017/S1478951520000814

[16] Duan Y, Huang J, Yu R, Lin F, Liu Y. Evaluation of the effect of death education based on the peace of mind tea house: a randomized controlled trial of nursing trainees at Xiamen University, China. BMC Nurs. 2024;23(1):597. 39183284 10.1186/s12912-024-02188-1 PMC11346280

[17] Yang Y, Li Y, Fu J, Guo D, Xue J. Effectiveness of a four-stage death education model based on constructivist learning theory for trainee nursing students. J Multidiscip Healthc. 2025;18:1371–80. 40066250 10.2147/JMDH.S500169 PMC11892376

[18] Cohen L, Manion L, Morrison K. Action research. In: Research methods in education. Routledge; 2017. p. 440–56.

[19] Holter IM, Schwartz Barcott D. Action research: what is it? How has it been used and how can it be used in nursing? J Adv Nurs. 1993;18(2):298–304. 8436721 10.1046/j.1365-2648.1993.18020298.x

[20] Johnson ES. Action research. In: Oxford Research Encyclopedia of Education. 2020.

[21] Cornish F, Breton N, Moreno-Tabarez U, Delgado J, Rua M, De-Graft Aikins A, et al. Participatory action research. Nat Rev Methods Primers. 2023;3(1):34.

[22] Wong PT, Reker GT, Gesser G. Death Attitude Profile—Revised: A multidimensional measure of attitudes toward death. In: Death anxiety handbook: Research, instrumentation, and application. Taylor & Francis; 2015. p. 121–48.

[23] Hao Y, Zhan L, Huang M, Cui X, Zhou Y, Xu E. Nurses’ knowledge and attitudes towards palliative care and death: a learning intervention. BMC Palliat Care. 2021;20(1):50. 33765995 10.1186/s12904-021-00738-x PMC7993469

[24] Frommelt KHM. The effects of death education on nurses’ attitudes toward caring for terminally ill persons and their families. Am J Hosp Palliat Med. 1991;8(5):37–43. 10.1177/104990919100800509 1742142

[25] Bugen LA. Coping: Effects of Death Education. In: The Final Transition. Routledge; 2019. p. 366–76.

[26] Povedano-Jimenez M, Granados-Gamez G, Garcia-Caro MP. Work environment factors in coping with patient death among Spanish nurses: a cross-sectional survey. Rev Latino-Am Enfermagem. 2020;28:e3234. 10.1590/1518-8345.3279.3234 PMC7164927 32321038

[27] Zheng R, Bloomer MJ, Guo Q, Lee SF. New graduate nurses’ coping with death and the relationship with death self-efficacy and death anxiety: a multicentre cross-sectional study. J Adv Nurs. 2021;77(2):795–804. 33145826 10.1111/jan.14621

[28] Wu S, Li Y, Ye Y, Wu Y. Impact of continuous nursing based on WeChat Public Platform and Sojump on fluid exchange in patients with peritoneal dialysis. Chin J Pract Nurs. 2021;37(4):248–55.

[29] BALA J. Contribution of SPSS in Social Sciences Research. Int J Adv Res Comput Sci. 2016,7(6):250–4.

[30] Kleinheksel AJ, Rockich-Winston N, Tawfik H, Wyatt TR. Demystifying content analysis. Am J Pharm Educ. 2020;84(1):7113. 32292185 10.5688/ajpe7113 PMC7055418

[31] Byrne D. A worked example of Braun and Clarke’s approach to reflexive thematic analysis. Qual Quant. 2022;56(3):1391–412.

[32] Munten G, Van Den Bogaard J, Cox K, Garretsen H, Bongers I. Implementation of evidence-based practice in nursing using action research: a review. Worldviews Evid Based Nurs. 2010;7(3):135–57. 19778316 10.1111/j.1741-6787.2009.00168.x

[33] Radbron E, McCance T, Middleton R, Wilson V. Maintaining momentum in action research. Nurse Res. 2021. 10.7748/nr.2021.e1789. 10.7748/nr.2021.e1789 34196513

[34] Wu Q, Zhu P, Ji Q, Shi G, Qian M, Xu H, et al. The effect of death education course utilizing constructivist learning theory on first grade undergraduate nursing student attitudes and coping abilities towards death: a mixed study design. Nurse Educ Today. 2023;126:105809. 37058871 10.1016/j.nedt.2023.105809

[35] Albanese MA, Mitchell S. Problem-based learning: a review of literature on its outcomes and implementation issues. Acad Med. 1993;68(1):52–81. 8447896 10.1097/00001888-199301000-00012

[36] Liu JL, Xu YJ, Xia Y, et al. Progress in the application of card games in cancer patients. Shanghai Nurs. 2025;25(2):67–70.

[37] Yoong SQ, Schmidt LT, Chao FFT, Devi KM, Wang W, Zhang H. Nursing students’ perspectives and learning experiences of participating in a palliative and end-of-life care simulation programme: a qualitative study. Nurse Educ Today. 2024;134:106103. 38277759 10.1016/j.nedt.2024.106103

[38] Beddoe AE, Murphy SO. Does mindfulness decrease stress and foster empathy among nursing students? J Nurs Educ. 2004;43(7):305–12. 15303583 10.3928/01484834-20040701-07

[39] Fan JW, Schmidt LT, Chua MM, Lee GL, Goh LH, Lo CH, et al. The utility and feasibility of incorporating death cafes in undergraduate education: a qualitative exploration of medical and nursing students’ perspectives. Nurse Educ Today. 2025;145:106502. 39603210 10.1016/j.nedt.2024.106502

[40] Antonio GCB. Constructivism: an approach in training nursing students in the clinical setting. Int J Nurs. 2016;5(2):1–8.

[41] Wittenberg E, Ragan SL, Ferrell B, Virani R. Creating humanistic clinicians through palliative care education. J Pain Symptom Manage. 2017;53(1):153–6. 27876635 10.1016/j.jpainsymman.2016.11.004

