# Supplementary material for: Construction and implementation of a death education program for nursing interns: an action research study
Source: BMC Med Educ. 2026 Jan 7;26:195. doi: 10.1186/s12909-025-08495-8 (PMC12870230; doi:10.1186/s12909-025-08495-8)
Supplement: Supplementary file 1 — Supplementary Material 1. [file 12909_2025_8495_MOESM1_ESM.docx]

**Appendix A**

**Table A1 DAP-R scores before and after the first cycle of action research**

|  | Pre-teaching | Post-teaching | *t* | *P* |
| --- | --- | --- | --- | --- |
| Fear of death | 21.04±5.68 | 17.54±3.62 | 2.844 | 0.009** |
| Death avoidance | 15.29±3.50 | 11.92±2.24 | 3.479 | 0.002** |
| Neutral acceptance | 17.54±2.90 | 20.17±1.97 | -6.118 | < 0.001*** |
| Approach acceptance | 26.04±4.84 | 29.08±5.64 | -2.303 | 0.031* |
| Escape acceptance | 12.92±3.68 | 14.08±4.81 | -0.905 | 0.375 |

**Table A2 Comparison of FATCOD and CDS scores before and after the first cycle of action research (n=24)**

| Scale | Pre-teaching (Mean ± SD) | Post-teaching (Mean ± SD) | *t* | *P* |
| --- | --- | --- | --- | --- |
| FATCOD | 94.58±9.41 | 104.17±11.19 | -3.531 | 0.002** |
| CDS | 123.25±16.62 | 146.83±18.30 | -5.242 | 0.001** |

Footnote: FATCOD = Frommelt Attitude Toward Care of the Dying Scale; CDS = Coping With Death Scale; * P < 0.05, ** P < 0.01, *** P < 0.001.
